# Reaching the sustainable development goals through healthy environments: are we on track?

**DOI:** 10.1093/eurpub/ckaa028

**Published:** 2020-05-11

**Authors:** F Racioppi, M Martuzzi, S Matić, M Braubach, G Morris, M Krzyżanowski, D Jarosińska, O Schmoll, D Adamonytė

**Affiliations:** c1 World Health Organization European Centre for Environment and Health, Bonn, Germany; c2 World Health Organization Asia-Pacific Centre for Environment and Health, Seoul, Republic of Korea; c3 World Health Organization Regional Office for Europe, Copenhagen, Denmark; c4 WHO Collaborating Centre on Natural Environments and Health, European Centre for Environment and Human Health, University of Exeter Medical School, Exeter, UK; c5 Environmental Research Group, School of Population Health and Environmental Sciences, Faculty of Life Sciences and Medicine, King’s College London, London, UK

## Abstract

The adoption of the 2030 Agenda for Sustainable Development in 2015 opened new opportunities to work towards healthy environments through ‘whole of government’ and ‘whole of society’ approaches. It created a strong policy platform that acknowledges health as a result and an enabler of sustainable policies across all sectors of government. Five years into the process, an initial analysis of emerging trends indicates that, despite some encouraging developments in policy as well as overall progress in economy and technology, there remains a gap between rhetoric, ambition and reality. In particular, the monitoring system for environment and health-related sustainable development goals (SDGs) and targets requires further development; inequalities in environment and health persist and in some areas have increased; equity is not yet a central element of implementation and reporting on the achievement of the SDGs; and, most worrying of all, trends in key environmental indicators that are vital to the survival of the human species, such as those related to climate change and biodiversity, are still on an overall negative path. In summary, governments must significantly and rapidly increase action to secure the habitability and safety of planet Earth. The public health community assumes an unprecedented role in placing and maintaining health and equity at the heart of the political agenda. This demands new governance models conferring on the health sector a clear mandate and legitimacy to operate across sectors. It also requires enhancing capacities among health professionals to embrace this new level of complexity, understand the multiple links between sectoral policies and health, and successfully engage with other government sectors and stakeholders.

## Introduction

The adoption of the 2030 Sustainable Development Agenda and its sustainable development goals (SDGs) in 2015 represents a historical milestone for public health in general, and for environment and health in particular. For the first time, and across sectoral boundaries, health is explicitly recognized and understood as both a necessary condition for sustainable development, and at the same time as its outcome, with the underlying aspiration of ‘leaving no one behind.’^1^ Also, for the first time, it is recognized that the SDGs are universally applicable, and represent a commitment for all countries, regardless of their level of income. This universality aspect is a major departure from the ‘traditional’ international development agenda, normally understood as mostly applicable to developing countries, as for example embodied by the Millennium Development Goals.^2^

This ambitious and visionary shift makes all countries responsible for the collective achievement of the SDGs. It results from a better understanding of how human species relate to planet Earth, involving the notion that humans must live within specific limits or ‘planetary boundaries’ related to biophysical sub-systems and processes in order to keep the Earth hospitable. According to recent research, it is possible to identify nine planetary processes, which are fundamental to Earth-system functioning, each of which is being modified by human actions. These include climate change, novel entities, stratospheric ozone depletion, atmospheric aerosol loading, ocean acidification, biochemical flows, freshwater use, land-system change and biosphere integrity. For four of these processes, such boundaries had already been crossed by 2015: climate change; the loss of biodiversity; the addition of phosphorous and nitrogen to crops and ecosystems; and land use changes, including deforestation. This breaches what is termed a ‘safe operating space for humanity’, not least because the affected systems, which are in various ways connected, are very likely to respond in a non-linear fashion, and possibly reach tipping points of no return. In particular, climate change and biosphere integrity have been identified as ‘core boundaries,’ because each of them has the potential on its own to drive the Earth system into a new state should they be substantially and persistently transgressed.^3^^,^^4^ The notion of planetary boundaries is a powerful game-changer, as it links the fate of each nation and of the future generations to the actions and policies of all the other nations, making all countries equally accountable for the survival of the human species.

There are profound implications, globally and in Europe, starting with the necessity to understand and accept that the damage to global ecosystems, may alter their capacity to provide vital benefits. These include the provision of food and water, regulation of climate, floods, disease, wastes and water quality, soil formation, photosynthesis and nutrient cycling and cultural services.^5^ In a world connected environmentally, economically and socially, environmental change elsewhere will impact on our own health through, e.g. migration or food insecurity.^6–8^

In addition, there is still a long way to go, globally and in Europe, before well-established risk factors related to air pollution, water and sanitation, hazardous chemicals, and noise are successfully addressed. Taken together, in the WHO European Region these risk factors still account for some 1.4 million deaths per year and are responsible for around 26% of ischaemic heart disease, 25% of strokes and 17% of cancers in Europe.^9^ Nearly half of this burden is attributable to outdoor and indoor air pollution, with mortality rates twice as high in low- and middle-income countries of the Region (84 deaths/100 000 population) as compared with high-income countries (42 deaths/100 000 population). Air pollution has also emerged as one of the leading risk factors for non-communicable diseases, and it is estimated to account for more than 20% of deaths attributable to cardiovascular diseases.^10^ At the same time, over 16 million people still lack access to basic drinking-water; more than 31 million people need basic sanitation and 14 diarrhoea deaths a day can be attributed to inadequate water, sanitation and hygiene, with rural areas, poor people and children under 5 years being most affected, particularly in the Caucasus and central Asia.^11^

Five years into the SDG process, some reflection on progress seems appropriate, focusing on an analysis of those SDGs most closely related to environment and health (see table 1).

**Table 1:** SDGs of particular relevance to environment and health

SDG 3	Good health and well-being
SDG 4	Quality education
SDG 6	Clean water and sanitation
SDG 7	Affordable and clean energy
SDG 8	Decent work and economic growth
SDG 10	Reduced inequalities
SDG 11	Sustainable cities and communities
SDG 12	Responsible consumption and production
SDG 13	Climate action
SDG 14	Life below water
SDG 15	Life on land

Anticipating a time lag of, perhaps, 5–10 years to allow the effects of interventions to become visible in mortality and morbidity indicators, even ‘weak’ signals offer an opportunity to identify trends and suggest a path towards more concerted action. For these reasons, this article focuses on emerging trends in terms of policy environment, capacity to measure and monitor progress towards the achievement of the SDGs, trends in environmental health inequalities and trends in climate change and biodiversity, given their role as ‘core boundaries.’ Finally, the article urges the health community to embrace a much stronger role and leadership in stepping action up before it is too late.

## An evolution in health policies now favours progress towards the SDGs

Since the adoption of the 2030 Agenda for Sustainable Development in 2015, global and regional developments in policy have provided a solid foundation and a clear mandate that empowers and legitimates health sector actors to take an active role in the achievement of the SDGs (see table 2).

**Table 2:** Main global and regional policy frameworks of relevance to environment and health in the context of the 2030 Agenda for Sustainable Development

Policy frameworks	Year	Main contents
Ministerial Declaration of the Sixth Ministerial Conference on Environment and Health held in Ostrava, Czechia^15^	2017	Sets a commitment to ‘use the European environment and health process as an established intersectoral and inclusive process and platform for the implementation of the 2030 Agenda for Sustainable Development.’
WHO Thirteenth General Programme of Work (2019–24)^12^	2018	Aims at achieving the health-related SDGs making an impact in countries by ensuring healthy lives and well-being for all at all ages; achieving universal health coverage; addressing health emergencies and promoting healthier populations.
Declaration of the high-level meeting of the UN General Assembly on the prevention and control of non-communicable diseases^14^	2018	Recognizes ambient and household air pollution as one of the main risk factors for non-communicable diseases, alongside tobacco use, unhealthy diet, harmful use of alcohol and physical inactivity.
WHO Global Strategy on Health, Environment and Climate Change^13^	2019	‘This transformation requires focusing action on upstream determinants of health, environment and determinants of climate change in an integrated and mainstreamed approach across all sectors, enabled and supported by adequate governance mechanisms and high-level political will. The health sector needs to play a new role to drive this transformation, using a sustainable and equitable approach’.

In particular, the Ostrava Declaration identifies seven priority areas (air pollution; water, sanitation and hygiene; chemical safety; waste and contaminated sites; climate change; urban environments and sub-national action; and environmentally sustainable health systems) and commits Member States to develop national portfolios for action on environment and health, reflecting national priorities and needs.^15^

To ensure consistency with the SDGs framework, both the WHO Global Strategy and the Ostrava Declaration set out to monitor and report on progress towards their objectives using identical indicators to those established for reporting on the achievement of the SDGs and their targets.

In addition to enabling policies emerging in the wake of the 2030 Agenda for Sustainable Development, other contributory frameworks pre-date the SDGs. These include the European Health Policy for Health and Well-being (Health 2020),^16^ and multilateral environmental agreements, notably UN Conventions and Protocols, which address various environmental determinants of health, including air, water and sanitation, waste, chemical safety, industrial accidents and climate change.

## Monitoring progress towards the environment and health-related SDGs and targets is currently a challenge

With the intention of creating a supportive framework for Member States to monitor their progress towards reaching the SDGs and the targets most relevant to the Ostrava Declaration, the WHO European Centre for Environment and Health has recently reviewed the SDG monitoring system.^17^ The potential of supplementing its indicators by information collected in other existing systems of reporting was also analyzed. Important among these is the reporting system agreed under the Protocol on Water and Health to the Convention on the Protection and Use of Transboundary Watercourses and International Lakes (in 2016 Parties to the Protocol on Water and Health adopted a revised reporting mechanism in accordance with article 7 of the Protocol (http://www.unece.org/fileadmin/DAM/env/documents/2016/wat/11Nov_14-16_MOP4_PWH/Documents/ece_mp.wh_2016_4_english.pdf).), as well as other data collected by WHO and those reported under the European Union *acquis communautaire*. The ability of the indicators to reflect the equitability of actions was also assessed by evaluating availability of data on various population subgroups (males-females, urban-rural, rich-poor etc.).

The analysis identified 34 ‘key’ SDG indicators relevant to monitoring progress on the seven priority environment and health concerns identified in the Ostrava Declaration.^18^ However, data are only available for 19 of them and the indicators are not evenly distributed across environment and health priorities. Water and sanitation issues are best covered with seven indicators of which five have available data. In contrast, environmentally sustainable health systems are least covered, with only a single SDG indicator currently being developed. Data on most of the indicators identified in the WHO Global Strategy are available from at least some countries of the WHO European Region.

Additional work is necessary to further develop frameworks that allow the monitoring of progress towards global and regional targets relevant to environment and health and enable international comparisons. At the national level, Member States should be encouraged to develop monitoring methods that reflect national circumstances and needs while adopting the internationally recognized core set of indicators in environment and health.

## Despite the aspiration to ‘leave no-one behind,’ inequalities in environment and health persist, and in some areas have increased

Inequalities remain an abiding concern for the public health community everywhere. A key dimension of this is the unequal distribution of environmental hazards and risks, whether related to where people live, work, learn, play, seek care and spend their time or their individual circumstances.

The Second WHO Assessment Report on Environmental Health Inequalities reviewed the distribution of environmental risks within countries and quantified the inequalities in major health determinants related to urban, housing and working conditions, basic services and injuries.^19^ The report confirmed that environmental health inequalities persist and, in some cases, have become even stronger. In most cases, higher levels of environmental risk were found in disadvantaged population subgroups, which can be five times more exposed than other population groups. Inequalities in risks and outcomes occur in all countries in the WHO European Region, and the latest evidence confirms that socially disadvantaged population subgroups are not only more exposed to environmental hazards, but that environmental inequalities also contribute to health inequalities.^20^

Given the presence of environmental health inequalities across the WHO European Region, the ‘leaving no-one behind’ approach of the SDGs provides one of the most important commitments of national governments to both environmental justice and health equity. Yet, fulfilment of this commitment requires scrutiny in SDG implementation and the political will to identify disadvantaged groups of society and understand the causes.

This also requires making equity a more central element in SDG implementation and reporting, as shown by an initial assessment of the coverage of equity considerations in the environment-focused SDGs. This assessment was undertaken by the WHO European Centre for Environment and Health for this article, based on a qualitative screening of 38 recent Voluntary National Reviews on national SDG implementation^21^ submitted by Member States of the WHO European Region. For SDG 3 and SDG 10, the screening focused on the coverage of environmental equity topics within the national reports.

Overall, the findings suggest that most countries are aware of equity challenges when implementing and reporting SDG action: in 32 out of 38 national reports, at least one environmental equity dimension was mentioned.

However, there is a marked disparity between technical areas in which equity issues are being considered, with water, sanitation and hygiene representing the technical area in which most countries (29 out of 38) referred to equity challenges (table 3). This is followed by energy and housing, while air pollution and traffic injuries are at the end of the scale, suggesting that these may by largely considered general challenges for sustainable development and not (yet) a challenge for specific groups or areas only.

**Table 3:** Coverage of equity challenges by technical area (*n* = 38)

Environmental equity perspective	No. of national reports where the perspective was covered (%)
Water, sanitation and hygiene	29 (76.3)
Energy	20 (52.6)
Housing	16 (42.1)
Urban planning	11 (28.9)
Transportation	9 (23.7)
Access to green spaces	5 (13.2)
Regional development	3 (7.9)
Climate change	3 (7.9)
Environmental health	2 (5.3)
Air pollution	1 (2.6)
Traffic injuries	1 (2.6)

For the health-specific SDG 3, health equity dimensions were covered in some national reports only. The most frequently mentioned factors associated with health inequalities related to access to, and affordability of, health care services. Interestingly, environmental factors were only mentioned twice as drivers for health inequalities, representing only 5.3% of the reviewed national reports. A similar pattern emerged in the reporting on SDG 10, as environmental factors were scarcely mentioned as drivers of inequality. The most common topics covered in the relevant SDG 10 sections focused instead on income and economic inequalities, vulnerable populations and gender inequality issues.

Finally, the coverage of environmental equity varied greatly between reports. In some reports, there was a comprehensive overview of environmental inequalities, the most vulnerable population affected, and—for few countries—a description of future actions to decrease the inequalities. However, more commonly, the coverage of environmental equity perspectives remained descriptive, omitting a more analytical discussion of the social causes of inequality and the actions required to mitigate it.

Acknowledging the relevance of equity as a cornerstone of sustainable development, more effort is needed in many countries to assure that everyone benefits from environmental progress and no one is left behind.

## Trends in global change imply a mismatch between aspirations and reality

When looking at progress in terms of key global indicators, it appears that despite commitments and initial actions, some negative trends persist.

It is alarming, e.g. that key indicators of the impacts of climate change—such as sea level rise, ice loss and extreme weather events—increased during 2015–19, a period set to be the warmest 5-years on record according to the World Meteorological Organization.^22^ The report also states that the global average temperature has increased by 1.1°C since the pre-industrial period, and by 0.2°C compared with 2011–15. What is even more concerning, in the face of these negative trends, is that atmospheric greenhouse gas concentrations have also increased to record levels, locking in the warming trend for generations to come.

Particularly worrying is the acceleration of the changes and their impacts on health. For example, the 2019 report of the Lancet Countdown on health and climate change estimates that from 1990 to 2018, those aged 65 years and older in every region have become more vulnerable to heat and heatwaves, with Europe and the Eastern Mediterranean remaining the most vulnerable. In 2018, these vulnerable populations experienced 220 million heatwave exposures globally, breaking the previous record of 209 million set in 2015.^23^ The same report highlights that progress has been observed in some areas, such as greater share of renewable energy sources for power generation, increased resilience of cities and health systems to climate change and health adaptation spending. However, current progress in cutting greenhouse gas emissions is inadequate and warming is occurring faster than governments are able, or willing to respond.

In similar vein, the 2019 Report on Biodiversity and Ecosystem Services highlights that nature, across most of the globe, is now significantly altered by multiple human drivers.^24^ The great majority of indicators of ecosystems and biodiversity show rapid decline, and human actions threaten more species with global extinction now than ever before. The report also shows that, globally, local varieties and breeds of domesticated plants and animals are disappearing. This loss of diversity, including genetic diversity, poses a serious risk to global food security by undermining the resilience of many agricultural systems to threats such as pests, pathogens and climate change.

## The health community can play an important role to correct the current trajectory

Despite calls for ‘transformative’ and ‘cross-sectoral’ approaches, in reality the health community engagement and its capacity to influence the sectoral policies underpinning these global trends is arguably limited thus far.

The research community, often in close partnership with civil society organizations, has been rather effective in building evidence on the impacts of global changes, environmental hazards and health inequalities, and use it to advocate policies and interventions in pursuit of sustainable development. The Lancet Global Countdown on health and climate change, the Lancet Commissions on global health and pollution and health, and the Breathlife Campaign are sentinel examples.^25^

In parallel, however, the community of health professionals within governmental structures and health systems struggles to engage with other government sectors in influencing policies and decisions that profoundly affect health. For example, the lack of connection by health actors to climate change processes was indicated by countries responding to a recent WHO survey as one of the three main challenges faced in accessing international climate finance for health, along with lack of information on opportunities and lack of capacities to prepare country proposals.^26^ The reasons for this are multiple and complex, and include aspects related to governance and capacities.

Governance pertains to mandates, responsibilities and legitimacy in participating and contributing to strategic political discussions. For example, very few governments include representatives of the health sector in national delegations participating in the negotiation of global agreements on climate change. Arguably, this reflects a current failure of ministries of health to recognize climate change and health as a key element of their portfolio, as critically few governments have established effective mechanisms for multisectoral policy-making. At the same time, existing multisectoral policy platforms, such as the WHO European Environment and Health Process, the Protocol on Water and Health and the Transport, Health and Environment Pan-European Programme, provide opportunities for the health sector to engage with other sectors (such as transport, water management, agriculture and education) to strategize and tackle environmental risks in a multi-sectoral fashion.

Capacities pertain to the need to develop a public health workforce that understands not only the proximal threats to health and well-being that have been the ‘traditional’ targets of public health intervention, but also can prevent, counteract and contain more distal threats to health and well-being. Progress requires increased capacity among public health actors to understand these interconnections and embrace the complexity that this entails. They should be prepared and empowered to deploy growing scientific evidence to enhance their effectiveness in advocating and raising awareness of concepts such as ‘health in all policies’, ‘whole of government’ and ‘whole of society’ approaches to health.

This also requires enhanced capacity to understand and counter powerful commercial interests, and to critically appraise the health implications of major developments in technology. These include digitalization and the proliferation of new materials and chemicals. Moreover, the advocacy of more sustainable economic approaches such as transition from a linear economic model (based on the ‘take-make-use-dispose’ approach) to the ‘circular economy’ (based on restorative and regenerative approaches) is now essential. However, similarly to technological developments, this requires capacities to assess potential unintended risks to health, and to identify appropriate risk management strategies.

The health sector should thus develop its capacity, role and legitimacy to actively engage in the prospective assessment of policy developments and their implications. Improved monitoring systems, addressing also equity aspects, should support such involvement. This will take health actors beyond their traditional sphere of engagement, enabling their input to strategic economic decisions.^27^

Other important areas, where the health sector’s capacity needs to be enhanced relate to leadership and advocacy. This includes the need to collaborate with key non-governmental organizations and civil society on two fronts. The first is about highlighting the evidence on the urgency of action to protect the environment, and it is somewhat part of the established approaches to evidence-based advocacy. The second requires stepping out of the comfort zone of public health advocacy and pertains to the capacity of moving the hearts as well as the minds of the public and policy makers. One way of achieving this is the development of a more coherent and compelling narrative on the need to move from rhetoric and aspiration to profound system level change.^28^ However, this requires learning from and collaborating with political and social sciences, where these approaches are developed.

Governments should recognize, value and nurture a dialogue with actors in different sectors facilitating their understanding of their influence on health through strategic and operational decisions. Such a transformation demands a profound rethinking of, and investment in, training at the academic and professional level. Implicitly the targets of training must extend to a very broad professional constituency showing individual roles of various professions and benefits of the multisectoral collaboration.
